# Retrospective evaluation of plasma protein tumour markers for early lung cancer detection

**DOI:** 10.1038/s44276-024-00082-6

**Published:** 2024-08-19

**Authors:** Michael Peter Alan Davies, Ruwanthi Kolamunnage-Dona, Suzannah Phillips, Angela Lambert, Stephanie Tate, John Kirkpatrick Field

**Affiliations:** 1 Department of Molecular and Clinical Cancer Medicine, Institute of Systems, Molecular & Integrative Biology, The University of Liverpool, Liverpool, UK; 2 Department of Health and Data Science, Institute of Population Health, The University of Liverpool, Liverpool, UK; 3 Department of Biochemistry, Liverpool Clinical Laboratories, Liverpool University Hospitals National Health Service Foundation Trust, Liverpool, UK

## Abstract

**Background:**

Blood-based biomarkers might help lung cancer diagnosis. A panel of serum tumour markers (TM) has been validated for hospital referrals due to clinical suspicion of lung cancer. We have compared plasma from a cohort enriched for early-stage lung cancer, including controls from a healthy population cohort.

**Methods:**

TM assays for CEA, CYFRA 21-1, CA15.3, ProGRP and SCC were run on a Roche Elecsys 2010 Immunoassay Analyser for a retrospective, nested case-control cohort from the Liverpool Lung Project. The primary endpoints were the sensitivity and specificity of a pre-defined TM panel using published thresholds.

**Results:**

Except for ProGRP, TM levels were significantly higher in cases and ROC AUC values demonstrated significant discriminant power. Accuracy and levels were higher for late-stage cancers, except for ProGRP which was highest in stage 1 cancers. Although providing similar sensitivity (82.4% vs 88.5%), TMs performed worse for specificity (39.3% vs 82%) and overall (Youden’s Index 0.22 vs 0.77) and this was not improved by threshold optimisation or binary logistic regression.

**Conclusions:**

Although the TMs were associated with lung cancer status and discriminatory with a high sensitivity when combined, performance was compromised in early-stage disease, which casts some doubt on utility in the screening setting.

## Background

Lung cancer is a significant cause of premature mortality globally, being the 6th most common cause of death overall [1]. There were an estimated 2.09 million new cases of lung cancer and 1.76 million lung cancer deaths globally in 2018, representing almost 1 in 5 of all cancer deaths [2]. This high mortality is largely due to the late stage of the disease at diagnosis, which limits effective treatment options. As demonstrated by the NLST and NELSON low dose CT screening trials, earlier detection leads to reduced mortality [3, 4]. However, the definition of high risk based on age and smoking history needs to be substantially improved if screening programs are to maximise their positive impact on lung cancer early detection [5, 6]. Furthermore, low dose CT (LDCT) screening identifies indeterminate nodules in approximately 20% of individuals: too small to be considered for referral for invasive investigation or treatment, but carrying a significant risk of developing into invasive cancer [4, 6, 7]. New biomarkers are needed to identify those high-risk nodules and improve the overall specificity of lung cancer screening.

Accurate early diagnosis may therefore rely on improved minimally invasive biomarkers to (i) stratify those at high risk for further investigation (such as CT) in primary care or screening settings or (ii) manage indeterminate nodules found during CT investigations. Blood-based biomarkers offer great potential to fulfil both needs, as they can be administered in a primary care setting for risk stratification and provide more rapid answers that the current nodule management protocols that rely on repeat CT scans. Despite efficient rapid diagnosis by modern CT imaging techniques following primary care referral for suspected lung cancer, blood-based biomarkers would also benefit earlier lung cancer diagnosis outside of screening, especially for those individuals often excluded due to a lower perceived risk (e.g. light or never smokers).

Panels of proteins or protein-derived tumour markers (TM) found to be significantly higher in cancer patients may provide TM for use in diagnosis aiding early detection (if highly sensitive) or nodule management (if providing additional specificity alongside CT). We have therefore tested a panel of TM using an existing clinically validated platform (Elecsys, Roche Diagnostics) for their ability to differentiate known cancer cases from controls, either individually or in a diagnostic multi-TM signature. The TM panel in question has previously been validated in serum by Molina et al. [8]: CEA, CYFRA 21-1, CA15.3, proGRP, SCC, and NSE. We have tested five of these TM in plasma; the Elecsys NSE assay is only suitable for serum, so was excluded. Our retrospective case-control cohort includes samples from lung cancer cases (enriched for early stage disease) and matched controls with long-term follow-up (from both diagnostic hospital clinics and a healthy high-risk population); this cohort was selected in order to help assess the suitability of the TM panel in a population more closely resembling the lung cancer screening setting.

## Methods

### LLP cohort

Samples, risk questionnaires and clinical data from Liverpool Lung Project (LLP) participants [9] were obtained following voluntary informed consent, in accordance with the Declaration of Helsinki. Ethical approval was obtained from the Liverpool Central Research Ethics Committee (ref 97/141). Lung cancer cases were identified through NHS Digital (now NHS England) via the National Cancer Registration and Analysis Service or through case note review. Individual level healthcare data is available only for restricted purposes and must be governed by a data sharing agreement, so is only available upon request. The LLP lung cancer risk score (LLPv3) was calculated based on questionnaire data [10].

Cases (Table 1) were selected from those with the most common histological subtypes (49% adenocarcinoma, 43% squamous cell carcinoma and 8% small cell carcinoma). For increased relevance to the LDCT screening population early-stage cancers were enriched in the cohort (53% stage IA - IIB, 14% IIIA, 33% IIIB-IV). All samples will have been taken within 3 months of confirmed diagnosis of primary lung cancer and prior to any treatment.

**Table 1:** Histology and stage distribution of lung cancer cases.

Histology	TNM stage version 7 (% within histology)				Total
	IA-IB	IIA-IIB	IIIA-IIIB	IV	
Adenocarcinoma	86 (48.6%)	15 (8.5%)	28 (15.8%)	48 (27.1%)	177 (48.0%)
Squamous cell carcinoma	49 (30.4%)	28 (17.4%)	44 (27.3%)	40 (24.8%)	166 (43.6%)
Small cell carcinoma	5 (16.1%)	3 (9.7%)	12 (38.7%)	11 (35.5%)	31 (8.4%)

We have determined that based on a fixed prevalence of 50% (from 1:1 case:control ratio) and an expected sensitivity and specificity of 95%, if we set the lower 95% confidence limit to be greater than 88.5% as defined by Molina et al., 185 cases would be required to provide 95% power.

For each case, sex and age matched control subjects were identified, with similar smoking duration; controls were also matched for storage time, i.e. how long samples had been stored frozen, (Table 2, Supplementary Fig. 1). Sex was defined as sex at birth, reported by subject or taken from medical records. Ages given are ages when the plasma samples were taken. LLPv3 risk scores were calculated on questionnaire data available closest to date plasma taken, if necessary, age and smoking duration were adjusted accordingly. The majority of control samples (*n* = 318, 86%) were taken in a population setting (population controls, PC), relatively healthy individuals with no suspicion of lung cancer. Fifty-one controls (14%) were recruited in hospital clinics; 14 in a COPD clinic (hospital control COPD, HC-COPD) and 37 in a lung cancer diagnostic clinic (hospital control lung cancer, HC-LC), but all controls were cancer-free with a median follow up time of 4.8 y for PC, 4.75 y for HC-COPD and 3.65 y for HC-LC. The requirement for a minimum follow-up time to define cancer-free controls excluded samples with the shortest storage times (Supplementary Fig. 1F).

**Table 2:** Characteristics of cases and controls.

Characteristic	Case	Control	Statistic
Sex: male	212	212	Chi-Square 0 *P* = 1
female	157	157	
Age (mean years, s.d.) (median, IQR)	68.1 (8.8) 68 (62–75)	68.0 (8.8) 68 (61–75)	Mann–Whitney *P* = 0.94 Skewness −0.2
Age group: 30–49 years	5	5	Chi-Square 0.09 *P* = 0.99
50–59 years	56	55	
60–69 years	142	145	
70–79 years	135	132	
80–89 years	31	32	
Smoking status: Current	166	168	Chi-Square 0.221 *P* = 0.90
Former	192	188	
Never	11	13	
Total	369	369	
Smoking duration(years) mean (s.d.)	40.2 (14.9)	39.8 (14.7)	Mann–Whitney *P* = 0.63
Median (IQR))	43 (33, 51)	43 (33, 50)	Skewness −1.0
Pack-years mean (s.d.)	41.5 (23.1)	38.0 (23.4)	Mann–Whitney *P* = 0.010
Median (IQR)	39.8 (25.4, 54.0)	34.5 (22.0, 49.0)	Skewness 1.1
Quit-years mean (s.d.)	10.6 (16.9)	11.3 (16.9)	Mann–Whitney *P* = 0.58
Median (IQR)	1 (0, 16)	3 (0, 19)	Skewness 16.9
LLPv3 risk score mean (s.d.)	3.40 (2.92)	3.71 (2.98)	Mann–Whitney *P* = 0.045
Median (IQR)	2.65 (1.33, 4.55)	3.12 (1.61, 5.03)	Skewness 1.8
Storage time (years) mean (s.d.)	11.7 (4.3)	12.2 (4.0)	Mann–Whitney *P* = 0.014
Median (IQR)	10 (8, 16)	10.1 (8.8, 15.7)	Skewness 0.5
CA15.3 (kU-L) mean (s.d.)	28.7 (38.2)	19.7 (10.4)	Mann–Whitney *P* = 3 × 10^−5^
Median (IQR)	20 (15, 29)	18 (12, 25)	Skewness 9.4
CEA (ng-mL) mean (s.d.)	24 (140)	3.3 (2.4)	Mann–Whitney *P* = 4 × 10^−16^
Median (IQR)	4.0 (2.7, 9.1)	2.9 (2.0, 4.0)	Skewness 19.4
CYFRA21.1 (ng-mL) mean (s.d.)	7.0 (19.5)	1.9 (1)	Mann–Whitney *P* = 0
Median (IQR)	2.5 (1.7, 5.6)	1.7 (1.2, 2.4)	Skewness 13.3
ProGRP (pg-mL) mean (s.d.)	117 (329)	51.3 (26.1)	Mann–Whitney *P* = 0.18
Median (IQR)	47.3 (35.6, 67.3)	46.9 (36.0, 59.0)	Skewness 8.2
SCC (ng-mL mean (s.d.)	2.7 (5.1)	1.3 (1.0)	Mann–Whitney *P* = 2 × 10^−5^
Median (IQR)	1.3 (0.9, 2.3)	1.2 (0.8, 1.7)	Skewness 7.8

EDTA plasma samples from LLP subjects were collected by standardised protocols (between 1998 and 2017), with a single cell depletion centrifugation (2200 × *g*, 15 min.) prior to storing at −80 °C, samples underwent a further cell depletion spin after thawing and prior to analysis. Prior to 2011 samples were collected in Sarstedt Monovette EDTA tubes, which were stored at 4 °C before being spun after transfer to the laboratory. During 2011 blood collection was swapped to using Greiner K2EDTA Gel Vacuette (Part No. 455040), with a local spin prior to transfer to the lab.

### Assays

Assays and associated reagents were provided by Roche Diagnostics: CEA (assay 11731629322, standard 11731645322), CYFRA21-1 (assay 11820966122, standard 11820974322), SCC (assay 07028253190, standard 7126999190), ProGRP (assay 06505961190, standard 6505988190), CA15.3 (assay 03045838122, standard 03045846122), Diluent MultiAssay (3609987190), PreciControl Tumor Marker (11776452122), Diluent Universal (11732277122). Assays were run on a Roche Elecsys 2010 Immunoassay Analyser at Liverpool Clinical Laboratories, with the lab blinded to the lung cancer status. Paired cases and controls were randomised for analysis across multiple assay runs (batches of 80). Intra- and Inter-run controls were included as per standard hospital laboratory protocols and TM values were reported as absolute concentrations.

### Statistical analysis

The primary endpoint was the sensitivity and specificity of a pre-defined TM panel in a retrospective case-control cohort. Molina et al. published results from a large series of consecutive hospital-referred patients [8] and achieved a sensitivity 88.5%, specificity 82% for TM scored as positive if any single TM was above the threshold they defined previously (with individual TM sensitivities of 17.1–56.5% and specificities of 93.5–97%). We judge the accuracy achieved in our cohort against these values. Secondary endpoints include: assessment of confounding factors (e.g. other diseases, pre-analytic factors); assessment of additional benefit from combining biomarkers with clinical factors and LLP risk score.

We determined, based on a fixed prevalence of 50% (from 1:1 case:control ratio) and an expected sensitivity and specificity to 95%, if we set the lower 95% confidence limit to be greater than 88.5% defined by Molina et al. [8], 185 cases would be required to provide 95% power.

TM levels are reported as values, median, and interquartile range or as proportions above/below a defined level (including thresholds [8] defined previously). TM levels were compared using nonparametric [Wilcoxon, Mann–Whitney U (MW), Kruskal–Wallis (KW)] or parametric tests (Student’s *t* test), as appropriate (all tests 2-sided, no adjustments for multiple comparisons). Proportions between groups were compared using the chi-square test. Sensitivity, specificity and accuracy were calculated using standard formulas. Receiver operating characteristic (ROC) curves were constructed to assess diagnostic performance, compare accuracy of different TM panels and determine optimal cut-points for dichotomisation. Logistic Regression models were tested as an alternative to simplistic positive/negative scoring. Multivariate analysis was used to explore the optimal model, based on the initial 5 TM and addition of alternative variables.

## Results

### TM score accuracy of predicting lung cancer status

**Fig. 1:**
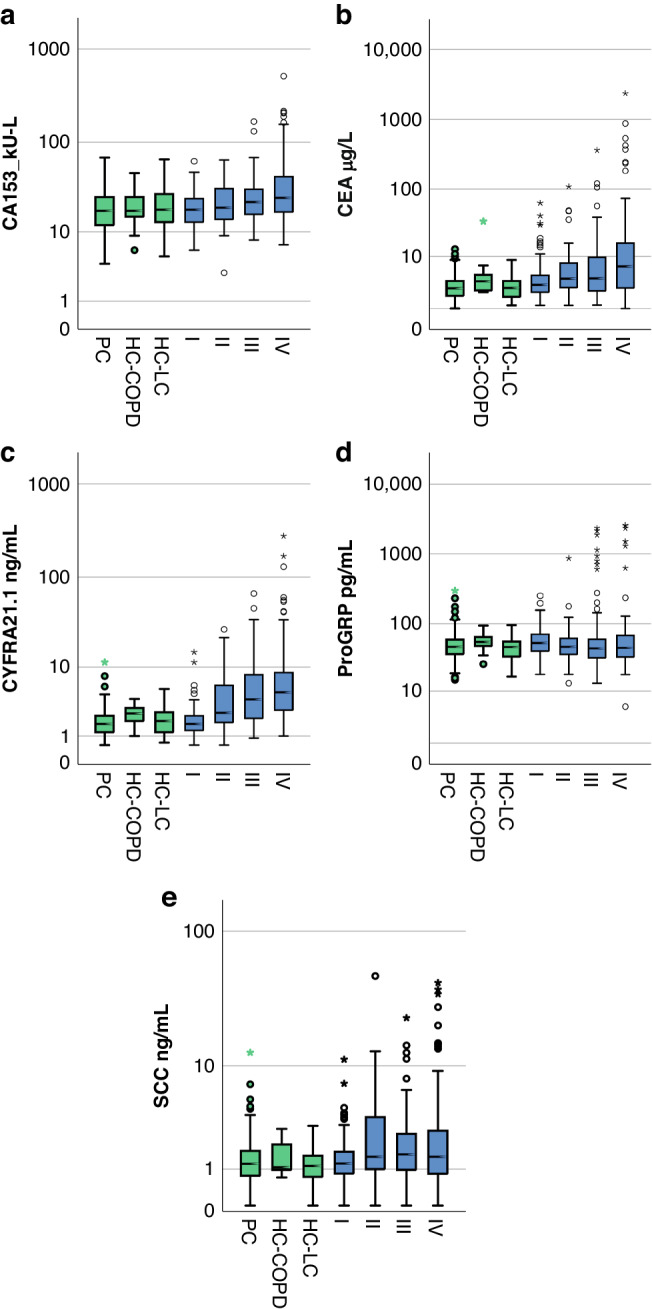
Association of TM levels with tumour stage (I–IV) and type of control. TM levels (CA153 (**a**), CEA (**b**), CYFRA21.1 (**c**), ProGRP (**d**) and SCC (**e**)) are shown for different control groups: (PC population cohort, HC-COPD hospital cohort query COPD, HC-LC hospital cohort query lung cancer) and for different stages of cancer.

In the selected LLP case-control cohort, TM levels (Table 2, Fig. 1) were higher in cases than controls for CA15.3 (MW *P* = 3 × 10^−5^), CEA (MW *P* = 4 × 10^−16^), CYFRA21.1 (MW *P* = 0) and SCC (MW *P* = 2 × 10^−5^), although not significantly so for ProGRP (MW *P* = 0.18). Using the thresholds defined by Molina et al. [8] (Table 3), the proportion of positive cases (i.e. sensitivity) was between 15.2% (CA153) and 46.1% (ProGRP); the specificities ranged between 58.3% (ProGRP) and 94.6% (CA153). For CA153, CEA and CYFRA21.1 we found lower sensitivity and specificity in our case-control cohort than in the hospital diagnostic setting of Molina et al. [8]. For ProGRP, and SCC we found a higher sensitivity, but again lower specificity, especially for ProGRP.

**Table 3:** Accuracy of TM prediction for lung cancer and association with tumour stage and histology.

		CA15.3	CEA	CYFRA21.1	ProGRP	SCC	Any TM
AUC		0.585	0.674	0.699	0.528	0.592	NA
AUC CI		0.544–0.627	0.635–0.713	0.661–0.737	0.486–0.517	0.550–0.633	
AUC P		<0.001	<0.001	<0.001	0.19	<0.001	
Cut-point		35 kU/L	5 µg/L	3.3 ng/mL	50 pg/mL	2 ng/mL	NA
% Positive controls		5.4	13.3	6.8	41.7	15.5	60.7
% Positive cases		15.2	37.7	39.0	46.1	30.1	82.4
Chi Square P		<0.001	<0.001	<0.001	0.24	<0.001	<0.001
Sensitivity LLP (Molina et al.)		15.2 *(25.1)*	37.7 *(56.5)*	39.0 *(56.1)*	46.1 *(17.1)*	30.1 *(20.7)*	82.4 *(88.5)*
Specificity LLP (Molina et al.)		94.6 *(97.0)*	86.7 *(93.5)*	93.2 *(96.1)*	58.3 *(95.2)*	84.5 *(97.8)*	39.3 *(82.0)*
Youden’s Index LLP (Molina et al.)		0.10 *(0.22)*	0.24 *(0.50)*	0.32 *(0.52)*	0.04 *(0.12)*	0.15 *(0.19)*	0.22 *(0.71)*
Accuracy LLP		54.9	62.2	66.1	52.2	57.2	60.8
% Positive	Stage I (*n* = 140)	3.6	23.6	10.0	52.9	15.7	69.3
	Stage II (*n* = 46)	17.4	37.0	41.3	37.0	43.5	78.3
	Stage III (*n* = 84)	17.9	36.9	57.1	39.3	40.5	92.9
	Stage IV (*n* = 99)	28.3	58.6	63.6	46.5	35.4	93.9
Chi Square *P*		<0.001	<0.001	<0.001	0.13	<0.001	<0.001
NSCLC % positive		15.4	37.3	40.8	42.9	32.0	81.7
SCLC % positive		12.9	41.9	19.4	80.6	9.7	90.3
Chi Square *P*		0.71	0.61	0.019	0.00005	0.0096	0.23
NSCLC AUC (*P*)		0.58 (<0.001)	0.68 (<0.001)	0.71 (<0.001)	0.50 (0.93)	0.60 (<0.001)	NA
SCLC AUC (*P*)		0.61 (0.03)	0.58 (0.13)	0.59 (0.11)	0.82 (<0.001)	0.48 (0.74)	NA
LLP optimum	Cut-point	17.5 kU/L	5.9 µg/L	3.25 ng/mL	67.2 pg/mL	2.4 ng/mL	
	Sensitivity	63.6	35.4	40.9	25.3	23.4	86.9
	Specificity	49.6	91.9	92.4	84	92.4	30.8
	Youden’s	0.13	0.27	0.33	0.09	0.16	0.18
	Accuracy	56.6	63.4	66.7	54.7	57.8	59.1
96% specificity	Cut-point	38.5 kU/L	7.15 µg/L	3.75 ng/mL	96.15 pg/mL	2.88 ng/mL	
	Sensitivity	13.3	29.3	36.0	11.1	19.3	63.9
	Specificity	95.4	95.5	95.1	95.9	95.9	80.1
	Youden’s	0.09	0.25	0.31	0.07	0.15	0.44
	Accuracy	54.4	62.2	65.6	53.6	57.6	71.9

**Fig. 2:**
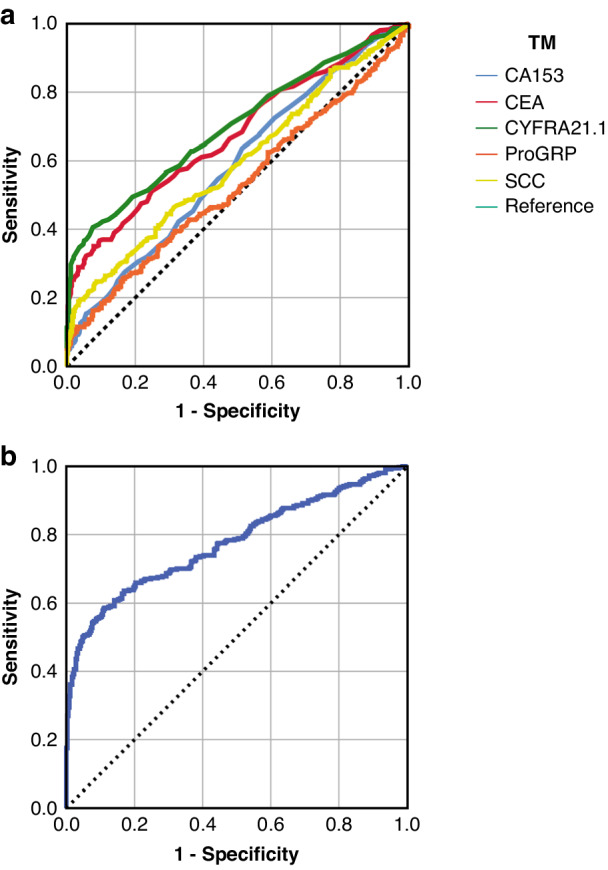
ROC analysis in the whole case-control cohort. ROC AUC graphs for each TM (**a**) and for logistic regression model based on LLP data (**b**).

When taking the combined TM scores, with any positive TM counting as positive, the sensitivity in the LLP cohort (82.4%) almost matched that of Molina et al. (85.5%), but the specificity was much lower (39.3% vs 82.0%) as was the Youden’s Index (0.22 vs. 0.71). Nevertheless, except for ProGRP, each TM was predictive of lung cancer status by ROC analysis with AUC ranging from 0.585 for CA15.3 to 0.699 for CYFRA21.1 (Table 3, Fig. 2).

The proportion of positive cases was not significantly different between SCLC (*n* = 31) and NSCLC (*n* = 343) for CA15.3, CEA or combined TM (Table 3). However, CYFRA21.1 (40.8% vs 19.4%, MW *P* = 0.019) and SCC (32.0% vs 9.7%, MW *P* = 0.0096) were more often positive in NSCLC and ProGRP significantly more often positive in SCLC (80.6% vs 42.9%, MW *P* = 0.00005). It is therefore likely that ProGRP contributes to the overall effectiveness of the combined TM panel by increasing sensitivity for SCLC. This is also demonstrated by the fact that in ROC analysis for SCLC ProGRP had an AUC of 0.82 (95% CI 0.71–0.93, *P* = 7 × 10^−9^) and was the only TM where the AUC value was greater for SCLC than NSCLC (Supplementary Fig. 2C).

**Fig. 3:**
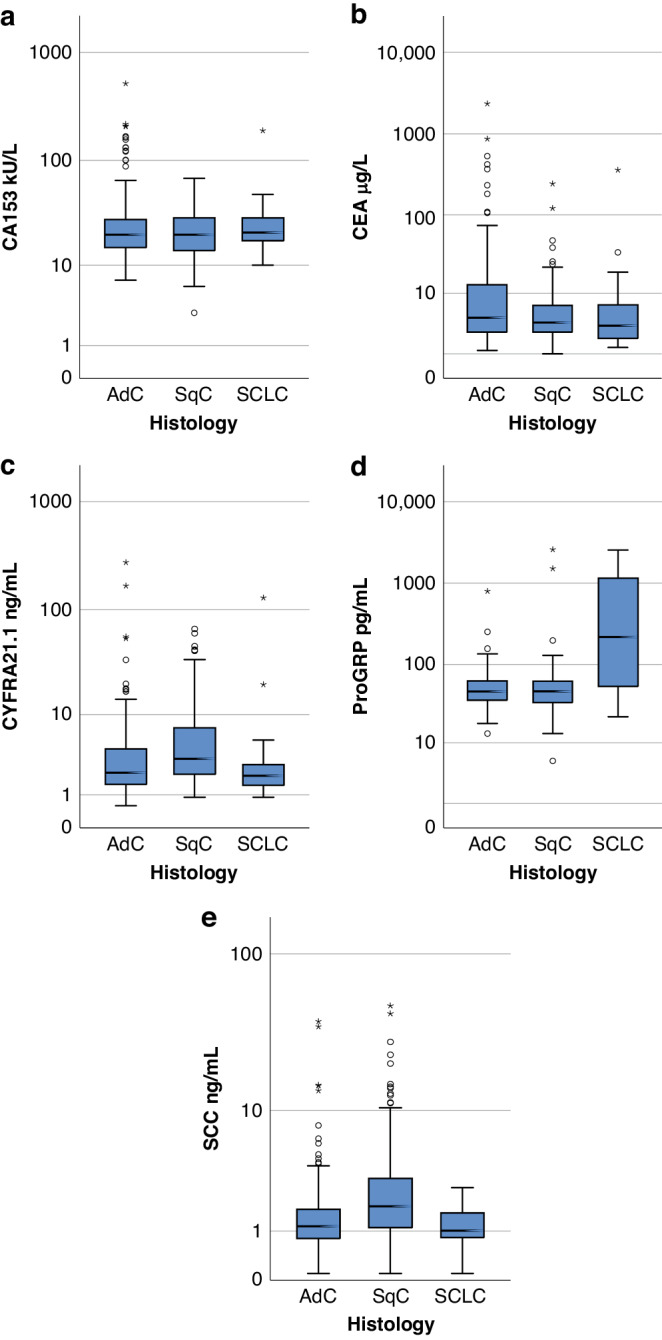
TM levels in cancer cases of different histology. Boxplots for TM levels in adenocarcinoma (AdC), squamous cell carcinoma (SqC) and small cell lung cancer (SCLC) for CA153 **a**, CEA **b**, CYFRA21.1 **c**, ProGRP **d** and SCC **e**.

When considering differences in TM levels within NSCLC (Fig. 3), CYFRA21.1 was significantly associated with histology (Chi-Square *P* = 0.002): higher in SqC than Adc (Fig. 3c: MW *P* = 0.00002) and NSLC compared to SCLC (MW *P* = 0.028); as was SCC (Fig. 3e: Chi-Square *P* = 0.002, MW *P* = 10^−8^ AdC vs SqC, 0.028 NSCLC vs SCLC). ProGRP (Fig. 3d) was no different between AdC and SqC (MW *P* = 0.82), but was significantly higher in SCLC (MW *P* = 10^−8^) and overall associated with histology (Chi-Square *P* = 0.0003). CEA had a relatively weak association with histology (KW *P* = 0.036) being slightly higher in AdC, but no significant association with histology were seen for levels of CA153.

### TM scores for different disease stage

Whilst all TM except ProGRP (AUC 0.515) exhibited significant discriminatory accuracy in late-stage disease (up to AUC 0.869 for CYFRA21.1; Supplementary Table 1, Supplementary Fig. 2A), the discriminatory power was significantly lower in early-stage disease (Supplementary Fig. 2B). For stage I lung cancers, despite being the largest group (*n* = 198), only CEA (AUC 0.615 *P* = 0.00001) and CYFRA21.1 (AUC 0.576 *P* = 0.003) maintained significance. Most TM were highest in late-stage disease (Fig. 1), with significantly higher levels in late-stage (III-IV) compared to early stage (I-II) seen for CA153 (MW *P* = 0.0002), CEA (MW *P* = 4 × 10^−6^) and CYFRA21.1 (MW *P* = 0). Whilst 93.9% of late-stage disease were positive for any TM, only 75.9% of early-stage disease were, a significant difference (Chi-Square *P* = 0.00001). Notably, unlike other TM (Supplementary Fig. 3), the proportion positive was highest for ProGRP in the earliest stage (53%, Table 3, Supplementary Fig. 3D) and the association of TM levels with stage was not seen (Fig. 1); this was not due the association with SCLC, as most SCLC tested were late stage (Table 1). However, ProGRP [11] did not discriminate between cases and control when considering only stage I (Supplementary Fig. 2B; AUC 0.546 CI 0.494–0.598, *P* = 0.077).

### Potential confounding factors

Unlike the Molina study [8], which used sequential samples over a relatively short period with uniform collection process, this retrospective analysis used samples stored over a longer period and two different EDTA plasma tube types. Whilst longer storage times (>10 years) were matched between cases and controls, the storage time for cases tended to be shorter (Supplementary Fig. 2), due to the need to ensure that controls had sufficient follow-up to ensure they remained cancer-free; overall this was significant (MW *P* = 0.014). However, there is no clear pattern when looking at the relationship between storage time and individual markers.

Using the thresholds defined by Molina et al., logistic regression models were fitted for case/control for any TM being positive (TM score >1), including blood collection tube type, sample storage time and TM analysis batch as covariates in the model. Coefficients for storage time (z = −1.6, *P* = 0.11), tube type (z = 0.31, *P* = 0.75), or batch (z = −0.042 to −4.2, *P* = 0.67 to 1.0) were not significant compared to the combined TM score (z = 6.4, *P* < 10^−9^) implying that none are a potential confounding factor for the analysis of case/control status.

Cases and controls were well matched for age, sex, smoking status and smoking duration (Table 2, Supplementary Fig. 2). There was a minor difference in intensity of smoking (median 38.8 pack-years in cases compared to 34.5 pack-years in controls, MW *P* = 0.010), but no difference in the overall number of years quit (MW *P* = 0.58). The LLPv3 risk score was only marginally higher (MW *P* = 0.045) for selected controls (median 3.12%) compared to cases (median 2.65%), with the majority of both groups being considered high risk.

### Relationship between TM and smoking

Looking at TM levels in current, former and never smokers only CEA was not distributed the same across all groups (KW *P* = 0.0003). CEA was significantly higher (Supplementary Fig. 4A) in current than former smokers, in both cases and controls (*P* ≤ 0.0001), and higher in current than never smokers in controls (*P* = 0.0004). In controls, CEA had the strongest correlation with smoking duration (Pearson 0.24, *P* = 0.000006, Supplementary Fig. 4B), pack-years (Pearson 0.15, *P* = 0.0056) and years quit (Pearson 0.35, *P* = 10^−11^). Other TM did not exhibit the same relationship (Supplementary Fig. 4C–F).

### Differences within control groups

Although the majority of samples for controls were taken in the population setting (86%), there were some controls taken on hospital COPD clinics (3.8%) and lung cancer diagnostic clinics (10%). HC-LC controls tended to be younger than PC (*P* = 0.003) and with correspondingly lower smoking duration (*P* = 0.00003) and LLPv3 risk score (*P* = 0.00008). However, there was only minor difference in levels of some TM (Fig. 1): CA153 (KW, *P* = 0.82), CEA (KW *P* = 0.027; HC-COPD > PC, *P* = 0.21), CYFRA21.1 (KW, *P* = 0.36; HC-COPD > PC, *P* = 0.042), ProGRP (KW, *P* = 0.28), SCC (KW, *P* = 0.53). Nevertheless, the proportion of TM positive samples was highest for HC-COPD for CA15.3, CEA, and ProGRP (Fig. 2f, g, I); whilst for CYFRA21.1 the lowest proportion of positives was seen for PC (Fig. 2h).

### Optimised classification based on LLP data

Using cut-points giving the same specificity as reported by Molina et al. [8] (average of 96% across each TM individually), the sensitivities in the LLP cohort (Table 3) were significantly worse than in the diagnostic setting reported previously, except for SCC which was comparable (LLP 19.3%, Molina 20.7%).

To test if this was simply due to a shift in the signal between studies, we derived optimised cut-points for each assay, based on LLP data; there was some improvement in sensitivity for CA15.3 (63.6 vs 15.2%) and specificity for ProGRP (84.0 vs 58.3%), but no overall benefit, as seen in Youden’s Index values (Table 3).

Building a binary logistic regression model for quantitative LLP measurements of each TM (Supplementary Table 2) the TM contributing most were CYFR21.1 (odds ratio 1.46, *P* = 10^−8^) and CEA (odds ratio 1.18, *P* = 10^−7^). The logistic regression model gave an AUC of 0.776 (CI 0.742–0.811, *P* = 10^−37^) and based on a cut point at the maximum Youden’s Index of 0.473, the accuracy achieved is 73.6% (sensitivity 58.3%, specificity 89.0%). At a defined specificity of 82% (as achieved by Molina et al. [8] for the “Any TM” classifier) the LLP logistic regression cut point achieves a sensitivity of 63.6% (compared to 82.4% achieved by Molina et al.).

Hence, choice of cut-points optimised on LLP data does not significantly improve accuracy of TMs, but does support the use of multiple biomarkers for improved accuracy.

## Discussion

Using a retrospective, nested case-control cohort we have demonstrated that a panel of TM previously validated at the time of diagnosis [8] can be used to classify lung cancer with a reasonable sensitivity. This is despite the fact that we have used EDTA-plasma rather than serum for our testing and have used retrospective, frozen samples. That the TM are relatively robust in frozen plasma may allow their use in a wider range of both clinical and research studies.

However, in our cohort, selected to include a greater proportion of early-stage disease and with controls matched for three of the major lung cancer risk factors (age, smoking duration and sex), the TM have significantly less discriminatory power, with lower sensitivity. This is true using the same quantitative thresholds for each TM, thresholds optimised on our own data or thresholds chosen to recapitulate the 96% average specificity demonstrated previously. The lower sensitivity for individual TM might be due to the use of EDTA as an anticoagulant, but this would only be the case if plasma measurements under-reported levels. Actually, the median for each TM in plasma was slightly higher in our frozen plasma samples than reported in fresh serum by Molina, and interquartile ranges overlap, except for ProGRP which was significantly higher (Table 2, median 46.9, IQR 36.0–59.0) than Molina reported (median 21, IQR 12–31 pg-mL).

The primary reason for this reduced sensitivity seems to be the inclusion of more early-stage disease than typically seen in diagnostic hospital clinics, such as that used previously. It is also noted that, as controls were matched for age and smoking duration, both cases and controls constitute high risk populations; the controls therefore resembling the target population for lung cancer screening. This is different from those receiving a negative diagnosis in the hospital setting tested previously [8], where patients with LC had a significantly higher smoking exposure history than those without it. Whist it might therefore be reasonable to suppose that part of the previously seen diagnostic accuracy was related to this smoking difference, we only found an association with smoking status or duration for CEA.

We have a higher proportion of early-stage lung cancer than Molina et al. (50% vs 24%). This was designed to better address the utility of the TM in a screening/early diagnosis setting, although admittedly early-stage cancers are even more common in LDCT screening (66.2% in the first 1 million scan in the US and 81.2% across five UK LDCT implementation studies) [12, 13]. This higher proportion of early-stage cancers has a significant impact of the lower sensitivity seen, as we show that the ability of the TM to classify cancers is greatly reduced in early-stage disease; this is not entirely unexpected, given the clear association with late-stage disease that we show for most individual TM and for any positive TM, and that larger tumours are likely to release more TM into the blood.

Nevertheless, for individual TM or “Any TM” in terms of accuracy in identifying lung cancer, our results broadly agree with those of Molina et al. We observed higher levels of specificity than sensitivity for individual TM but, as expected, the combined “any-positive” model improved sensitivity at the cost of lower specificity. Specifically, our data supports the conclusion that multiple TM offer greater accuracy that individual TM, but in the LLP data we saw a greater reduction in specificity (mean of individual TM 83.5% down to 39.2%, compared to a drop from 95.9% to 82.0% in the Molina study).

For SCC and ProGRP, the lower specificities in our cohort were associated with higher levels of sensitivity than found by Molina et al., but for CA153, CEA and CYFRA21.1 we found both lower sensitivity and specificity. We were unable to significantly improve discriminant accuracy either by defining individual new optimal thresholds or combining quantitative measurement in logistic regression models. This does not diminish the potential utility of the TM in the diagnostic setting where original thresholds defined were validated, although arguably current diagnosis was not improved in that setting. However, it does indicate that the different cohorts, rather than technical issues with optimal thresholds, underlie the reduced accuracy we find. The conclusion we draw is that the TM biomarker is not as powerful when applied to a different clinical setting, where most subjects are at high risk of lung cancer and a greater proportion of cases are early stage.

ProGRP was the only TM in our analysis that was not significantly higher in cases than controls and that failed to produce a significant AUC in ROC analysis. However, it was also the only TM that was not significantly associated with later stages of disease and was the only TM more frequently positive in SCLC than NSCLC (giving a significant AUC in SCLC). The inclusion of ProGRP in the panel may therefore be of particular benefit for SCLC and also help with early-stage detection. There is plenty of evidence that ProGRP (along with NSE, the TM we were unable to measure in plasma) are diagnostic biomarkers for SCLC [11] and other neuroendocrine tumours, but the apparent association with earlier stage is intriguing, especially as most SCLC tested were late stage. It is also worth noting that whilst normal range levels for other TM were slightly higher in our samples than previous serum measurements, for ProGRP plasma levels were significantly higher. Whether this apparent greater sensitivity for ProGRP in plasma is responsible for the different accuracy we report is unclear, but the failure to derive a better optimal threshold argues against it.

Molina describes utility of 6 TM (including NSE, which we excluded due to lack of a Roche assay) in 3144 consecutive individuals referred by their primary care physician because of the clinical suspicion of lung cancer (of which 1828 were confirmed as cases, 58%). However, it is a different setting to our case-control cohort, where we selected “healthy” controls having matched for gender, age and smoking history, but with the exception of a minority (10%) there was no suspicion of lung cancer at the time the plasma sample was taken. Hence, whilst Molina et al. address an important diagnostic setting, our cohort more closely resembles a screening cohort in terms of stage at diagnosis, with one important proviso: that we have matched controls for smoking, age and sex. Therefore, to be specific, it more closely resembles a high-risk screening cohort selected for LDCT, as by matching cases for age and smoking duration (the two major risk factors), we have selected controls that have similarly high risk (in fact slightly higher median and mean LLPv3 risk score). Our cohort does not reflect the screening population as a whole (before risk-based selection), so should not be used to infer anything about using TM for identifying those to be offered LDCT. Similarly, combining risk stratification tools, such as the LLP risk score, with TM in the current cohort offers little insight, as the primary benefit of the risk score is to exclude those of lowest risk and we have effectively done that in matching for three of the major risk factors.

Using logistic regression, we demonstrated that neither pre-clinical variables (such as storage time and analysis batch) nor lung cancer risk factors (such as age, or smoking duration) add significantly to the TM when building models for case status. We also showed that in our cohort the TM giving the highest odds ratios in a combined model were CYFRA21.1 followed by CEA. Despite matching to cases, our control cohort is broad enough to look at association between TM and potential confounding factors. The most notable association (seen both in cases and controls) was between CEA and smoking status, being higher in current smokers and those who smoked for longer. However, this did not impact the association between CEA and lung cancer status.

Given that we see evidence that the TM is associated with stage and (in the case of CEA) smoking, these are likely confounding factors contributing to higher sensitivity for Molina (they have no control over these in their consecutive series and smoking was clearly associated with case status, both in terms of status and mean pack years, *P* < 0.0001, Molina Table 1).

The limitations of this current study are primarily related to the study design and associated matching by age, smoking and gender. This limits our ability to look for association of TM with risk factors that are better represented in population or sequential cohorts, or to combine TM with other risk factors in a meaningful way. Specifically, with a prevalence based on 50:50, then the accuracy, Youden Index and AUC are virtually identical mathematically. However, population studies are both expensive and more susceptible to confounding if the biomarkers in question correlate with risk factors such as smoking; by matching for risk factors we largely eliminate such confounding. Notably only the relationship between CEA and smoking is strong enough to be evident within both cases and controls, but this does not confound the accuracy of CEA as a TM (indeed it was one of the strongest).

Sequential cohorts, such as that used by Molina, most closely resemble that particular diagnostic setting, and our study might therefore be considered inferior. However, we do not repudiate the earlier findings for diagnosis and, as we demonstrate, sequential cohorts will be more susceptible to acquisition bias, in this case for late-stage disease, and may not be as informative for other diagnostic setting (such as LDCT where a stage-shift towards earlier stage is achieved). Nevertheless, Molina did demonstrate some utility in nodule management (with high specificity across different nodule sizes, high negative predictive value in smaller nodule and high positive predicted value in larger nodules), which we were unable to recapitulate due to lack of CT data. Rather than in patients referred with symptoms, such studies might be best performed using plasma collected at baseline LDCT screening scan, especially where there is sufficient follow-up available [14].

There are many efforts to define biomarkers that may be of use in improving the early detection of cancer, with some of the most promising using highly multiplexed protein analysis [14–17]. However, whilst most of these focus on discovery and validation on research-only platforms, the current TM approach benefits from re-purposing clinically validated, commercially available, fully quantitative assays, in routine clinical laboratories. The approach of applying pre-determined thresholds and scoring for the above-threshold value of one or more TM, whilst simplistic (compared to the multi-parameter, relative expression models most often put forward from multiplexed discovery) is pragmatic and can achieve good sensitivity and specificity in the right setting.

The TM panel previously described has potential clinical utility to provide complementary information to the attending physician to better estimate the risk of LC presence in those attending hospital (to guide how aggressive the diagnostic strategy should be for the individual patient), not to substitute for the histologic diagnosis of cancer. Our results argue that this may be primarily true for late-stage disease, given that although specificity is largely retained sensitivity is compromised when the proportion of early-stage cancers is higher, as is the aim for early detection including LDCT lung cancer screening.

## Supplementary information

Supplementary Information

## Data Availability

The data and materials supporting the conclusions of this article are included in this published article (and its supplementary information files). Primary TM data and related sample metadata is available from the corresponding author on request, but individual level data can only be released under a suitable data sharing agreement due to informed consent restrictions.
