# Supplementary material for: Retrospective evaluation of plasma protein tumour markers for early lung cancer detection
Source: BJC Rep. 2024 Aug 19;2:59. doi: 10.1038/s44276-024-00082-6 (PMC11524055; doi:10.1038/s44276-024-00082-6)

Supplementary Information: Retrospective evaluation of plasma protein tumour markers for early lung cancer detection

Supplementary Table 1 ROC data for different stages (with number of cases).

Stage		CA15.3	CEA	CYFRA21.1	ProGRP	SCC
**Late**	**AUC**	0.651	0.750	0.869	0.515	0.620
**III-IV**	**AUC CI**	0.594 - 0.709	0.694 - 0.806	0.83 - 0.908	0.451 - 0.58	0.558 - 0.683
(n=127)	**AUC P**	10^{-7}	10^{-16}	10^{-35}	0.60	0.00006
**Early**	**AUC**	0.549	0.633	0.606	0.535	0.576
**I-II**	**AUC CI**	0.502 - 0.596	0.587 - 0.679	0.559 - 0.654	0.487 - 0.584	0.529 - 0.624
(n=233)	**AUC P**	0.045	10^{-8}	0.00001	0.15	0.0018
**Stage I**	**AUC**	0.536	0.615	0.576	0.546	0.554
(n=198)	**AUC CI**	0.486 - 0.586	0.564 - 0.665	0.525 - 0.628	0.494 - 0.598	0.503 - 0.604
	**AUC P**	0.16	0.00001	0.003	0.077	0.040
**NSCLC**	**AUC**	0.582	0.682	0.709	0.502	0.602
(n=330)	**AUC CI**	0.539 - 0.624	0.643 - 0.722	0.67 - 0.748	0.458 - 0.545	0.559 - 0.644
	**AUC P**	0.0002	10^{-16}	10^{-21}	0.93	10^{-6}
**SCLC**	**AUC**	0.619	0.584	0.589	0.818	0.482
(n=30)	**AUC CI**	0.524 - 0.713	0.458 - 0.71	0.474 - 0.703	0.71 - 0.927	0.377 - 0.586
	**AUC P**	0.031	0.13	0.11	6.9E-09	0.74

Supplementary Table 2 TM classification using binary logistic regression model (LR).

	B coef.	S.E.	Wald	LR P	Odds ratio
**CA15.3**	0.007	0.006	1.036	0.309	1.01
**CEA**	0.169	0.033	27.023	10^{-7}	1.18
**CYFRA21.1**	0.380	0.065	34.157	10^{-8}	1.46
**ProGRP**	0.006	0.002	6.241	0.012	1.01
**SCC**	0.178	0.082	4.696	0.030	1.19
**LR constant**	-2.513	0.283	78.872	10^{-18}	0.08

Supplementary Figure 1 Matching on subject characteristics (A) age at sample, (B) all smoking duration, (C) cigarette pack-years, (D) smoking quit-years, (E) LLPv3 risk score, (F) storage time.

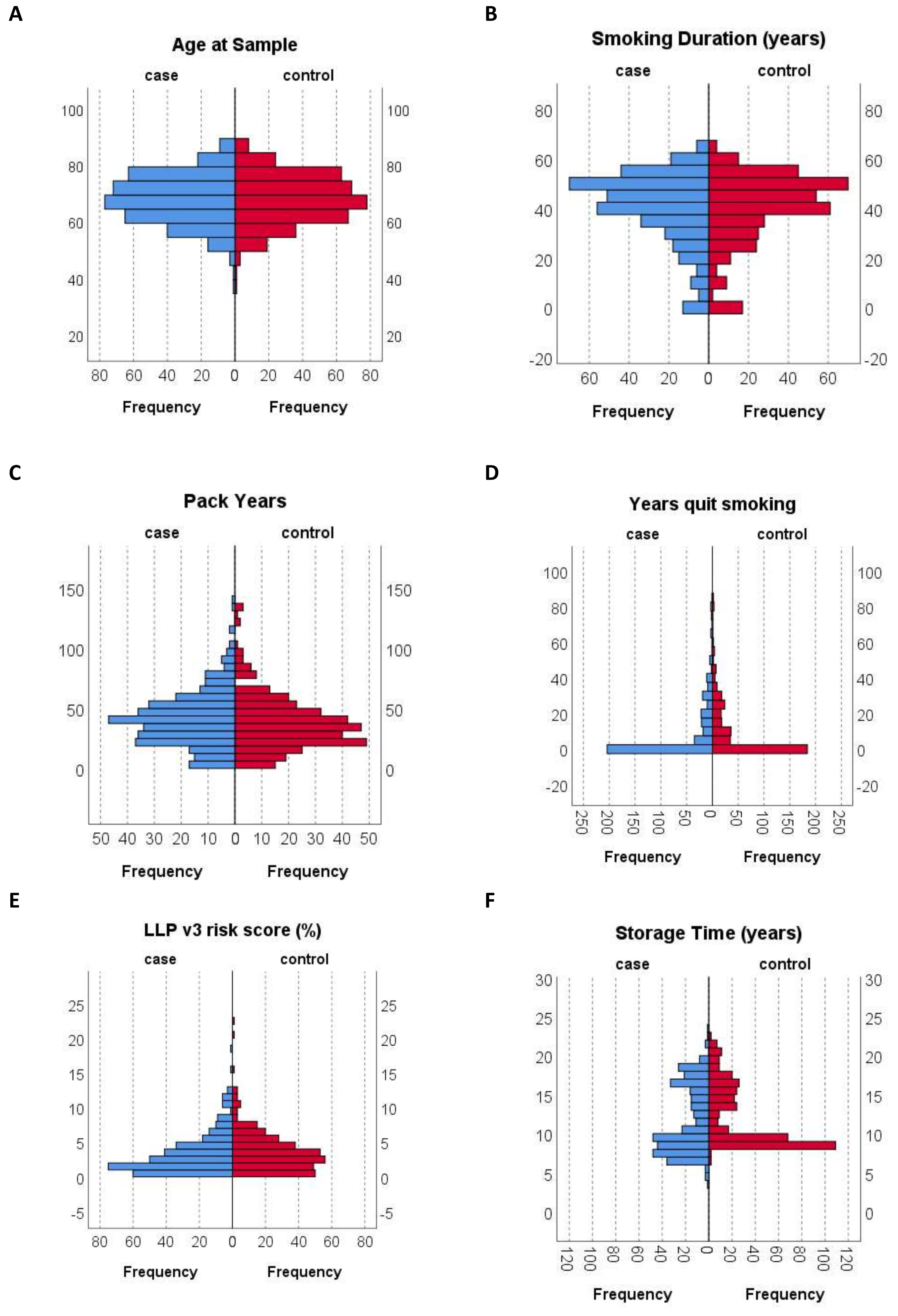

Supplementary Figure 2 ROC AUC graph for each TM in (A) late-stage disease only (127 cases), (B) early-stage disease only (233 cases), and (C) SCLC only cohort (31cases).

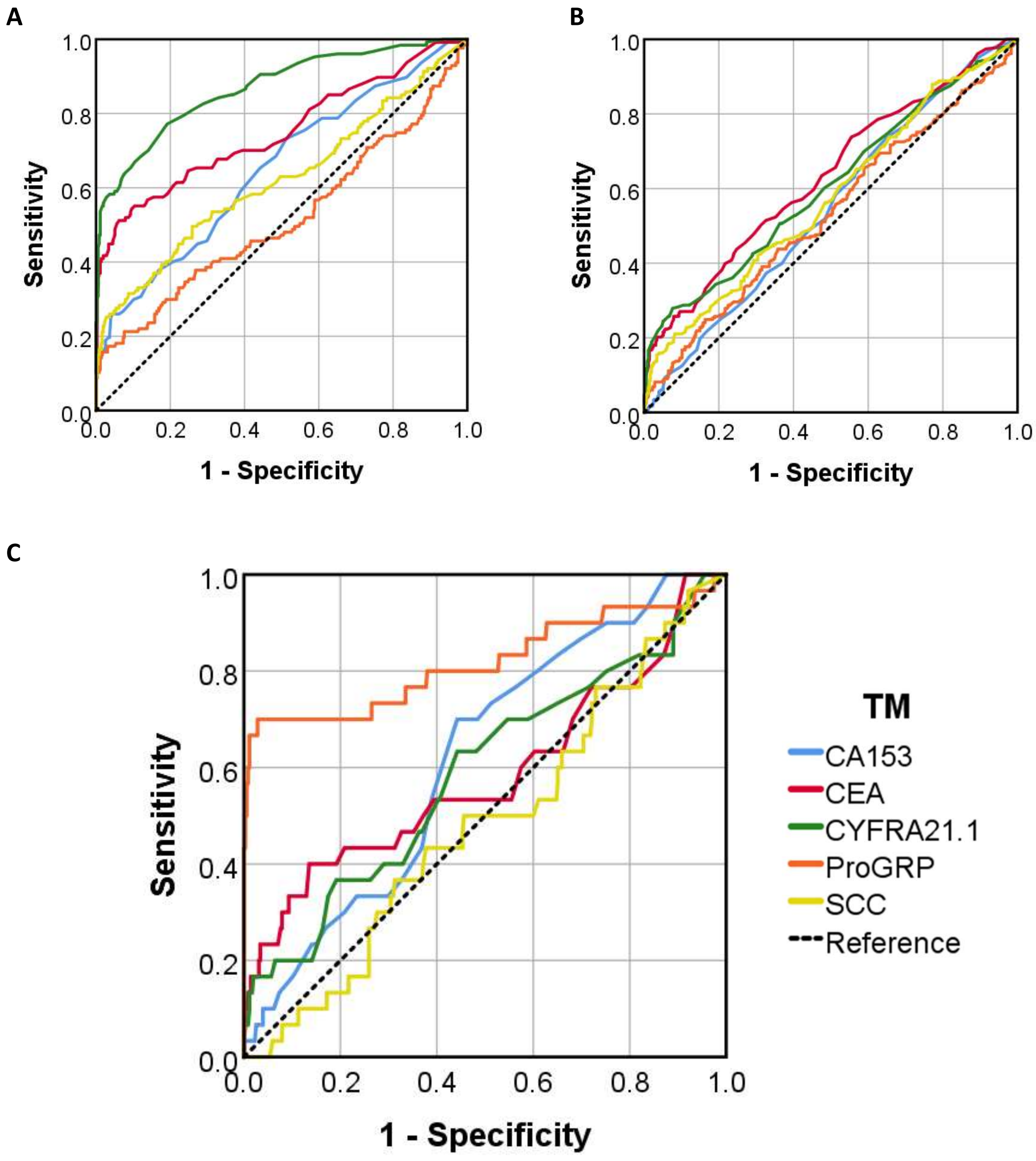

Supplementary Figure 3 Association of TM (% positive according to pre-defined cut-off) with tumour stage (I - IV) and type of control: PC= population cohort, HC-COPD = hospital cohort query COPD, HC-LC = hospital cohort query lung cancer.

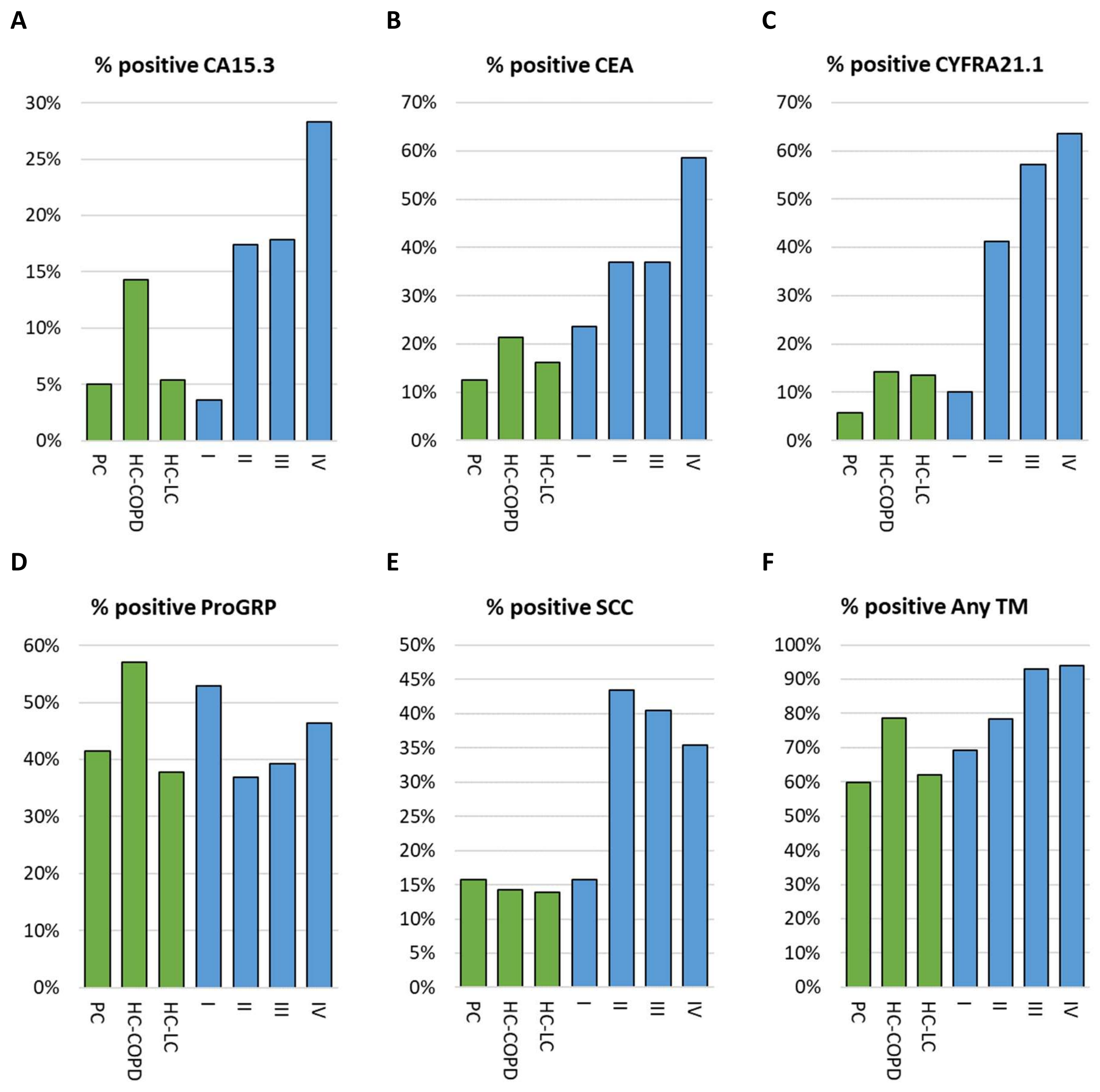

Supplementary Figure 4 TM associations with confounding factors. Association of CEA with smoking status in cases and controls (A) and correlation with smoking in controls (B), colour coded by smoking status. Association of CA153 (C), CYFRA21.1 (D), ProGRP (E) and SCC (F) with smoking status in cases and controls.

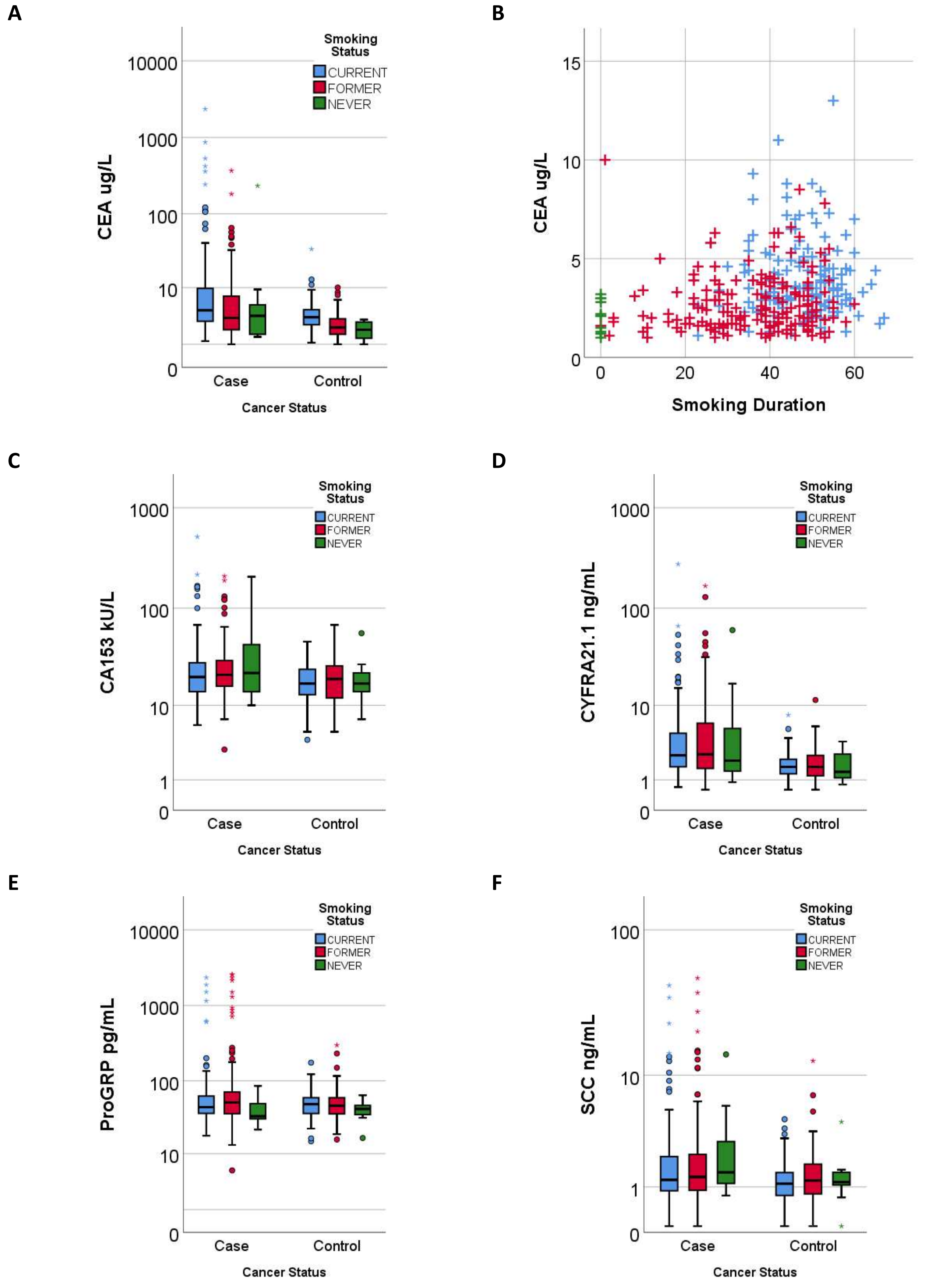

Supplement: Supplementary file 1 — Supplementary Information [file 44276_2024_82_MOESM1_ESM.pdf]
